# Transmission dynamics and attributable burden of tuberculosis among young and middle-aged adults in urban Shanghai: a genomic epidemiology study

**DOI:** 10.3389/fpubh.2026.1868508

**Published:** 2026-07-08

**Authors:** Shuang Zhang, Yangyi Zhang, He Zhan, Boshu Zhang, Sainan Zhang, Qing Su, Xiaofeng Cai, Yibiao Zhou, Jianhua Shi, Yuan Jiang, Meixia Yang

**Affiliations:** 1 Xuhui District Center for Disease Control and Prevention (Xuhui District Health Inspection Institute), Shanghai, China; 2 School of Public Health, Fudan University, Shanghai, China; 3 Shanghai Municipal Center for Disease Control and Prevention (Shanghai Academy of Preventive Medicine), Shanghai, China

**Keywords:** molecular epidemiology, population attributable fraction, student populations, transmission dynamics, tuberculosis, whole-genome sequencing

## Abstract

**Background:**

China remains a high tuberculosis (TB) burden country, and large urban centers such as Shanghai continue to face persistent transmission challenges driven by high population mobility. Whole-genome sequencing (WGS) provides high-resolution discrimination between recent transmission and reactivation; however, evidence at the district level remains limited. This study aimed to integrate WGS with epidemiological data to characterize local TB transmission dynamics in Xuhui District, Shanghai, and to identify demographic risk factors for recent transmission.

**Methods:**

A retrospective study was conducted among culture-positive pulmonary TB cases diagnosed between August 2021 and February 2025. WGS was performed for all available *Mycobacterium tuberculosis* isolates, and genomic clusters were defined using a threshold of ≤12 single-nucleotide polymorphisms. Epidemiological links were assessed through routine field investigations. Multivariable Firth penalized logistic regression and population attributable fraction (PAF) analyses were used to identify demographic predictors of recent transmission.

**Results:**

Among 170 genotyped patients, the Beijing lineage predominated (90.0%, 153/170). Fluoroquinolone resistance mutations were detected in 12.4% of strains. Demographic disparities in sex and age were observed between local and migrant residents (both *p* < 0.05). Six genomic clusters involving 17 patients (10.0%) were identified, and 50% of clusters contained at least one student case. Field investigations revealed close spatial proximity among clustered cases, including one pair residing within 50 meters. Multivariate analysis identified younger age as an independent correlate of genomic clustering. Compared with patients aged ≥45 years, the adjusted odds ratios for clustering were 42.75 (95% CI: 5.79–315.50) for those aged 15–24 years and 18.06 (95% CI: 3.76–86.73) for those aged 25–44 years (both *p* < 0.001). Notably, the joint PAF for individuals aged 15–44 years was 88.69%, with the 25–44-year age group accounting for the largest share of clustered cases (52.9%).

**Conclusion:**

Recent TB transmission in Xuhui District was predominantly driven by younger and middle-aged populations. Although students represent a high-risk subgroup, the broader working-age population contributed most to the overall transmission burden. These findings highlight the importance of integrating school-based interventions with community-level strategies targeting the active workforce in order to effectively interrupt transmission.

## Introduction

Tuberculosis (TB), caused by *Mycobacterium tuberculosis* (MTB), remains one of the leading causes of death from infectious diseases worldwide, with over 10 million new cases reported annually (1). As one of the 30 high TB burden countries identified by the World Health Organization (WHO), China contributes significantly to this global challenge. In 2023, China reported approximately 740,000 new TB cases, corresponding to an incidence rate of 52 per 100,000 population, which accounts for 6.8% of the global total (2).

At the city level, Shanghai has achieved a relatively low TB incidence, recorded at 19.2 per 100,000 in 2024 (3). However, persistent challenges remain for TB control, particularly among key populations such as migrant workers and students (4). High population mobility and dense social contact networks in urban environments facilitate TB transmission, while congregate settings like schools create favorable conditions for cluster outbreaks (5). These observations exemplify how socio-demographic factors—age, residency status, and educational or occupational settings—together with the structure of district-level health surveillance, shape who acquires and transmits TB even within low-incidence metropolitan areas. Disparities-focused, district-scale evidence integrating genomic and epidemiological data, however, remains scarce.

Xuhui District, located in the southwest of central Shanghai, serves as a quintessential model for studying TB dynamics in a high-density metropolitan environment. Covering an area of 54.76 km^2^, it has a permanent population of approximately 1.11 million, with an extremely high population density of over 20,000 persons per km^2^ (6). Notably, Xuhui is recognized as a premier educational and cultural hub, housing over 10 higher education institutions and numerous secondary schools, with a dense student population that is highly active in congregate learning and living environments. Furthermore, nearly one-third (approx. 31%) of the district’s residents are internal migrants (floating population, locally known as *liudong renkou*) (7). These demographic characteristics—high population density, a significant concentration of students, and substantial social mobility—create a complex interface for TB transmission between school-based clusters and the broader community.

Understanding TB transmission dynamics, particularly the distinction between recent transmission and endogenous reactivation, is crucial for tailoring effective public health interventions. Traditional genotyping tools such as MIRU-VNTR and spoligotyping offer limited resolution for differentiating between recent transmission and long-standing endemic strains (8, 9). Whole-genome sequencing (WGS), by contrast, enables high-resolution genotyping through the detection of single-nucleotide polymorphisms (SNPs), allowing for the fine-scale identification of recent transmission chains (10, 11). A threshold of ≤12 SNPs between isolates is widely accepted as indicative of recent transmission and has been applied in numerous international and domestic studies (12).

Despite advances in molecular epidemiology, most WGS-based TB studies in China have focused either on population-level transmission or on school outbreaks with confirmed epidemiological links. However, limited attention has been given to the potential overlaps between sporadic student cases and broader community transmission in urban settings, where high mobility and mixed social environments complicate TB control. Evidence regarding this overlap remains scarce, particularly at the district level.

Therefore, we conducted a retrospective molecular epidemiological study in Xuhui District, leveraging its unique status as a high-density educational hub. We integrated WGS with detailed epidemiological investigations to delineate local TB transmission patterns, identify key demographic risk factors for recent transmission, and examine the potential linking role of student cases between school and community settings.

## Methods

### Study design and participants

This retrospective study enrolled TB patients with culture-positive diagnoses from designated hospitals in Xuhui District, Shanghai, between August 15, 2021, and February 15, 2025. Clinical isolates from all eligible patients were referred to the TB Laboratory at the Shanghai Municipal Center for Disease Control and Prevention (Shanghai CDC) for centralized storage, species identification, drug susceptibility testing and WGS.

The following inclusion criteria were applied: (1) a diagnosis of TB at a designated hospital in Xuhui District within the study period; (2) residency or principal activity (e.g., employment or schooling) within Xuhui District, including commuters who worked or studied in Xuhui but resided in other districts, as well as those who lived in Xuhui but were diagnosed and notified by hospitals outside Xuhui District; (3) bacteriological confirmation (i.e., a sputum culture positive for MTB) (13); and (4) availability of a complete, viable, and traceable clinical isolate. Within this study population, migrants were defined as individuals without local Shanghai household registration (*hukou*), a classification routinely used in China’s TB surveillance system and previous molecular epidemiological studies to distinguish local residents from migrant populations (4, 14).

Conversely, patients were excluded if any of the following criteria were met: (1) infection with non-tuberculous mycobacteria (NTM); (2) culture contamination or WGS data that failed to meet quality control standards (as detailed in below); or (3) missing any of the following core epidemiological data—age, sex, household registration status, occupation/student status, treatment history, date of diagnosis, residential address (at the community level), or laboratory result records linked to the isolate.

### WGS and bioinformatic analysis

Clinical MTB isolates were obtained from the routine surveillance program of the Shanghai CDC. Genomic DNA was extracted from inactivated bacteria following established protocols (15). WGS was performed on qualified DNA samples using either the NovaSeq 6,000 or MGISEQ-2000 platform to generate 150-bp paired-end reads. Both sequencing platforms have been shown to generate highly concordant variant-calling results when standardized sequencing depth and quality-control criteria are applied (16–18). In this study, all samples were processed using a harmonized bioinformatics pipeline and identical SNP-calling parameters to minimize potential cross-platform variability.

WGS data were processed with a validated bioinformatics pipeline (14) for read alignment and variant calling. Samples with an average sequencing depth <20 × or genome coverage <90% were excluded. Strain lineages and genotypic drug resistance profiles were determined using TB-Profiler (v6.3.0) (19).

For molecular epidemiological analysis, a high-quality SNP dataset was constructed. Fixed SNPs (allele frequency ≥75%) were extracted from the whole-genome data, excluding those located in drug resistance-associated genes and repetitive regions (e.g., PPE/PE-PGRS families). A threshold of ≥75% was adopted following established TB whole-genome sequencing frameworks to distinguish fixed variants from low-frequency within-host microvariants while minimizing sequencing artifacts (12, 18, 20).

Pairwise SNP distances were calculated for all isolate pairs. Isolates with an SNP distance of ≤12 were considered genetically clustered, suggesting recent transmission.

Based on this SNP dataset, the GTR + GAMMA model was identified as the optimal nucleotide substitution model using ModelTest-NG (v0.2.0). A maximum-likelihood phylogenetic tree was then constructed with RAxML-NG (v1.2.2) (21), using 200 bootstrap replicates to assess nodal support. The tree was visualized and annotated with bacteriological data using the Interactive Tree of Life platform.^1^ Putative transmission links between strains were further inferred and confirmed using the R package Transcluster (22).

### Epidemiological investigation

A standardized initial epidemiological investigation was conducted for all patients by designated hospital staff at the time of admission or first visit. This investigation collected baseline demographic, clinical, and laboratory data, as well as information on close contacts. For patients linked to school settings, physicians from corresponding community health service centers performed additional, in-depth investigations. These investigations focused on tracing the patient’s activity trajectories from 3 months prior to diagnosis until 14 days after treatment initiation, with the goal of comprehensively identifying close contacts.

For patients grouped within the same transmission cluster, in-depth epidemiological investigation was conducted by a specialized team using a dual approach. First, self-reported contact histories were collected, including acquaintances, classmates, co-workers, and family contacts. Second, residential and workplace addresses and activity trajectories during the 6 months preceding diagnosis through 14 days after treatment initiation were systematically verified to identify spatial overlap among cluster members, independent of whether patients reported knowing each other. A confirmed epidemiological link required (a) mutual acquaintance with a clear history of contact prior to illness onset. In the absence of reported contact, (b) documented spatiotemporal overlap (e.g., residence in adjacent dwellings within the same community, or shared regular activity location) was treated as supportive evidence of a probable, but unconfirmed, link.

### Statistical analysis

Data management was performed using Microsoft Excel. Spatial relationships within clusters were depicted as schematic relative-distance diagrams without geographic coordinates. All statistical analyses were conducted using Stata version 14.0. Categorical variables were summarized as frequencies and percentages (%). Group comparisons were performed using Pearson’s Chi-square test or Fisher’s exact test, as appropriate. Fisher’s exact test was applied when expected cell counts were small or when the assumptions of the chi-square test were not satisfied, particularly for 2 × 2 contingency tables involving clustered cases.

To identify factors associated with strain clustering, univariate logistic regression analyses were first performed. Multivariable analysis was then conducted using Firth’s penalized logistic regression with *a priori* selected covariates based on epidemiological relevance and previous tuberculosis transmission studies, including age group, sex, student status, household registration status, and treatment history. This approach was chosen because of the limited number of clustered cases and sparse data in several categories, which could lead to small-sample bias and complete separation in conventional logistic regression models. Results are presented as adjusted odds ratios (aORs) with corresponding 95% confidence intervals (CIs). As a sensitivity analysis, a parsimonious Firth model including only age, sex, and student status was fitted to assess the robustness of the age effect.

## Results

### Characteristics of tuberculosis patients

**Figure 1:**
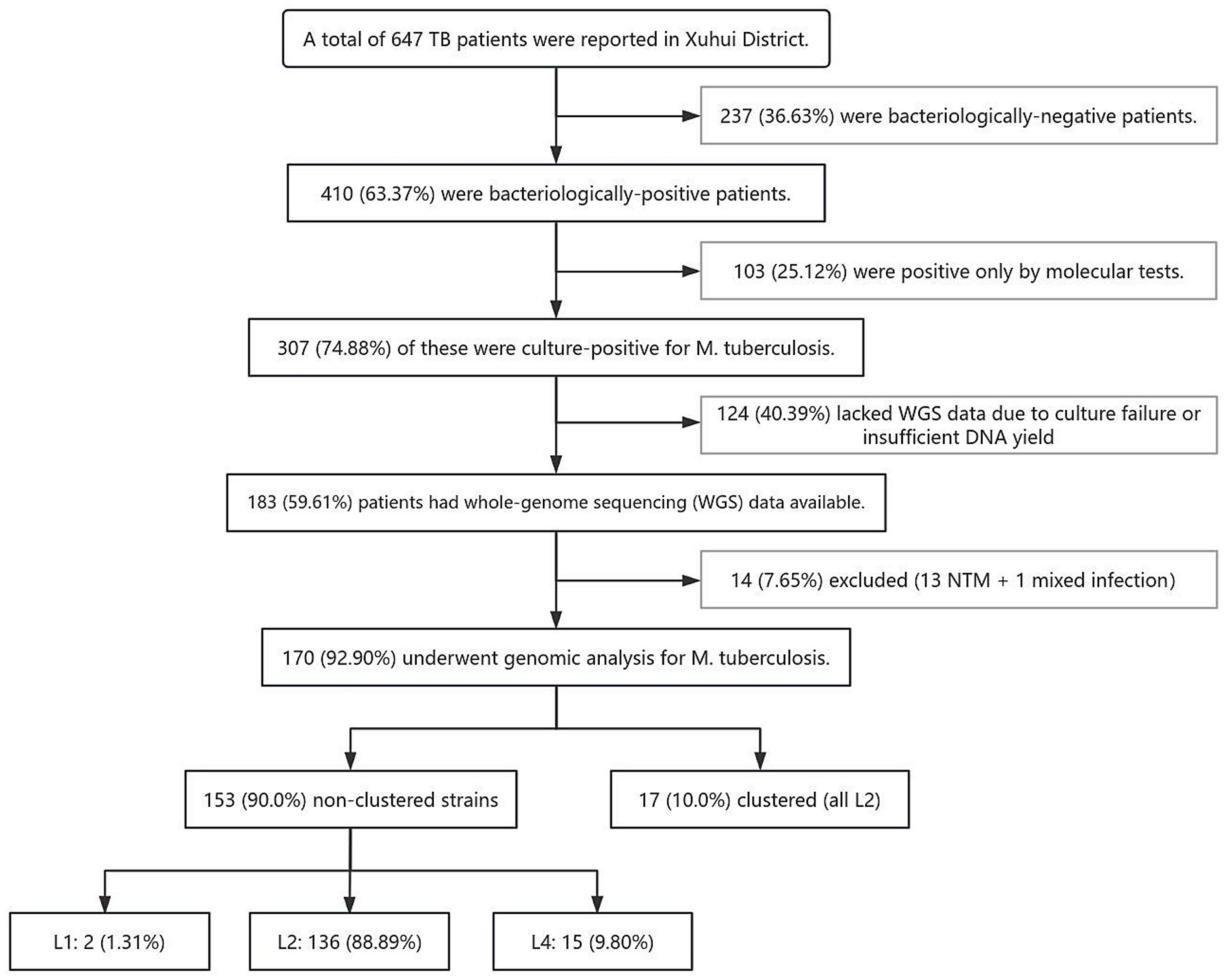
Flowchart of study population and sample selection. TB, tuberculosis; NTM, non-tuberculous mycobacteria; WGS, whole-genome sequencing. The flowchart illustrates the inclusion and exclusion criteria for patients with culture-positive *Mycobacterium tuberculosis*, including stratification by lineage (L1, L2, L4) and clustering status.

A flowchart of study population and sample selection is displayed in Figure 1. A total of 647 TB patients were reported in Xuhui District between August 15, 2021, and February 15, 2025. Among them, 410 (63.4%) were bacteriologically confirmed. After quality control of sputum culture and WGS data, 170 patients were ultimately included in the study. To assess potential selection bias related to the availability of viable isolates, we compared the baseline characteristics of the 170 included patients with those of the 471 reported TB patients in Xuhui District who had complete data but did not contribute a sequenced isolate (due to smear-negative status, culture failure, or non-availability of viable strains). As shown in Supplementary Table S1, the two groups were largely comparable in terms of age, sex, household registration, occupation, and treatment history (all *p* > 0.05), indicating that the included sample is broadly representative of the total notified population. Genomic clustering analysis identified 17 (10.0%) clustered cases, while the remaining 153 (90.0%) were classified as non-clustered cases.

The demographic and clinical characteristics of the 170 patients included in the study are summarized in Table 1. The cohort was predominantly male (64.7%, 110/170) and older, with most cases concentrated in the older age groups (45–64 years: 25.9%, 44/170; ≥65 years: 41.8%, 71/170). Most patients were new cases (92.9%, 158/170), and the predominant infecting strain was Lineage (L) 2, accounting for 90.0% (153/170) of cases.

**Table 1:** Demographic and clinical characteristics of tuberculosis patients.

Variable	Total	Household registration				Occupation			
		Migrant patients	Resident patients	*χ* ^2^	*p* value	Students	Non-students	*χ* ^2^	*p* value
	*n*	(*n* = 59)	(n = 111)			(*n* = 10)	(*n* = 160)		
Sex									
Male	110	30 (50.8)	80 (72.1)	7.60	0.006	5 (50.0)	105 (65.6)	–	0.326
Female	60	29 (49.2)	31 (27.9)			5 (50.0)	55 (34.4)		
Age in years									
15–24	16	8 (13.6)	8 (7.2)	41.80	<0.001	10 (100.0)	6 (3.7)	–	<0.001
25–44	39	27 (45.8)	12 (10.8)			0 (0.0)	39 (24.4)		
45–64	44	17 (28.8)	27 (24.3)			0 (0.0)	44 (27.5)		
≥65	71	7 (11.9)	64 (57.7)			0 (0.0)	71 (44.4)		
Treatment history									
New case	158	53 (89.8)	105 (94.6)	–	0.345	10 (100.0)	148 (92.5)	–	0.215
Retreatment case	12	6 (10.2)	6 (5.4)			0 (0.0)	12 (7.5)		
Strain lineage									
L2	153	52 (88.1)	101 (91.0)	0.35	0.555	9 (90.0)	144 (90.0)	–	1.000
Non-L2	17	7 (11.9)	10 (9.0)			1 (10.0)	16 (10.0)		
Clustering status									
Clustered	17	8 (13.6)	9 (8.1)	1.27	0.264	3 (30.0)	14 (8.8)	–	0.064
Un-clustered	153	51 (86.4)	102 (91.9)			7 (70.0)	146 (91.2)		
Drug resistance status									
DS-TB	124	41 (69.5)	83 (74.8)	Ref		7 (70.0)	117 (73.1)	Ref	
MDR/RR-TB	13	7 (11.9)	6 (5.4)	–	0.246	1 (10.0)	12 (7.5)	–	0.560
Other DR-TB	33	11 (18.6)	22 (19.8)	–	0.977	2 (20.0)	31 (19.4)	–	1.000

*Data are presented as n (%) unless otherwise specified. Intergroup comparisons were performed using the χ^2^ test or Fisher’s exact test, as appropriate. A dash (“–”) in the *χ*^2^ column indicates that Fisher’s exact test was used (e.g., when any expected cell count was <5 or for 2 × 2 tables with zero cell counts). Drug resistance status was categorized into three groups (DS-TB, MDR/RR-TB, and Other DR-TB) for analysis to avoid sparse expected cell counts and ensure the validity of statistical testing. Abbreviations: DS-TB, drug-susceptible tuberculosis; MDR/RR-TB, multidrug- or rifampicin-resistant tuberculosis.

Intergroup comparisons revealed significant associations between household registration and patient demographics. A higher proportion of males was observed in the local household registration group compared to the migrant group (*χ*^2^ = 7.60, *p* < 0.05). A significant difference in age distribution was also observed (*χ*^2^ = 41.80, *p* < 0.001). Specifically, locally registered patients were predominantly older adults, with individuals aged ≥ 65 years constituting 57.7% of this subgroup. In contrast, the migratory group was notably younger, with the 25–44 age group accounting for 45.8%. Additionally, significant differences were identified between student and non-student groups in age distribution (*p* < 0.05, Table 1). The genomic clustering rate was higher among students than non-students (30.0% vs. 8.8%), a difference that did not reach significance (*p* = 0.064, Table 1) and was no longer apparent after multivariable adjustment. This trend may reflect greater social mixing and mobility among students and warrants further investigation in larger cohorts.

### Clustering and spatial transmission analysis

**Figure 2:**
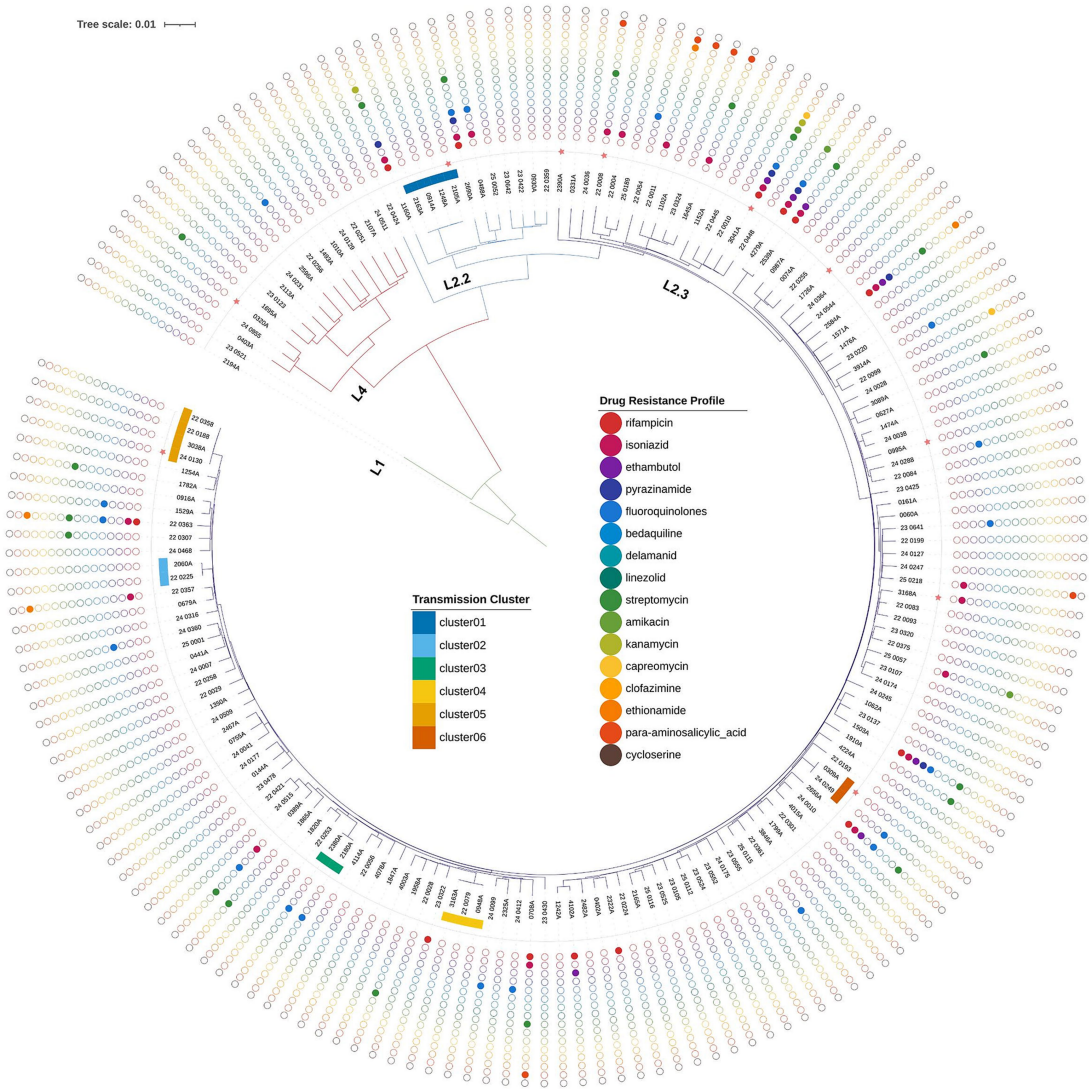
Maximum likelihood phylogenetic tree of 170 *Mycobacterium tuberculosis* isolates from Xuhui District, Shanghai (2021–2025). Branches are colored by lineage (L1, L2, L4); The inner color strips mark the six genomic transmission clusters; The outer rings show genotypic drug-resistance profiles for individual anti-TB drugs.

A maximum likelihood phylogenetic tree was constructed based on the WGS of 170 MTB clinical isolates collected in Xuhui District (Figure 2). Phylogenetic analysis revealed that the circulating strains predominantly belonged to three lineages. L2 (Beijing lineage) was dominant, accounting for 90.0% (153/170) of all isolates, followed by L4 (Euro-American lineage) at 8.8% (15/170) and L1 at 1.2% (2/170). Within the L2 strains, the modern Beijing sublineage L2.2.1 was predominant, comprising 68.8% (117/170) of the total collection.

Genomic analysis of the 170 clinical MTB isolates characterized the profile of drug resistance-associated mutations. Fluoroquinolone (FQ) resistance mutations were the most prevalent, identified in 12.4% (21/170) of strains, followed by isoniazid resistance in 11.8% (20/170) and rifampicin resistance in 7.6% (13/170). Among the 13 rifampicin-resistant (RR-TB) isolates, 10 were classified as multidrug-resistant TB (MDR-TB) and 3 as rifampicin-monoresistant TB. Notably, 6 MDR/RR-TB isolates also harbored fluoroquinolone resistance mutations (to levofloxacin or moxifloxacin) and met the definition of pre-extensively drug-resistant TB (pre-XDR-TB), accounting for 46.2% of all MDR/RR-TB isolates. No resistance-associated mutations to newer anti-TB drugs, including bedaquiline, delamanid, linezolid, and clofazimine, were identified in this cohort.

To assess recent transmission dynamics, we performed molecular clustering based on whole-genome SNP analysis. A total of 6 genomic clusters, comprising 17 strains, were identified using a pairwise SNP threshold of ≤12 to define recent transmission, resulting in an overall clustering rate of 10.0% (17/170). Cluster sizes ranged from 2 to 4 strains (Table 2; Figure 2). Notably, half of the clusters (3/6, 50%) included at least one student case. Epidemiological investigation revealed that in two of these clusters, non-student patients resided either in the same residential area as the student cases or in close proximity to their schools. This spatiotemporal overlap was consistent with the possibility of epidemiological linkage between school and community settings, although direct transmission pathways could not be confirmed.

**Table 2:** Strain clustering status of tuberculosis patients in Xuhui District.

Isolates per cluster	No. of clusters	Total isolates (%)
2	3	6 (35.3)
3	1	3 (17.6)
4	2	8 (47.1)
Total	6	17 (100.0)

**Figure 3:**
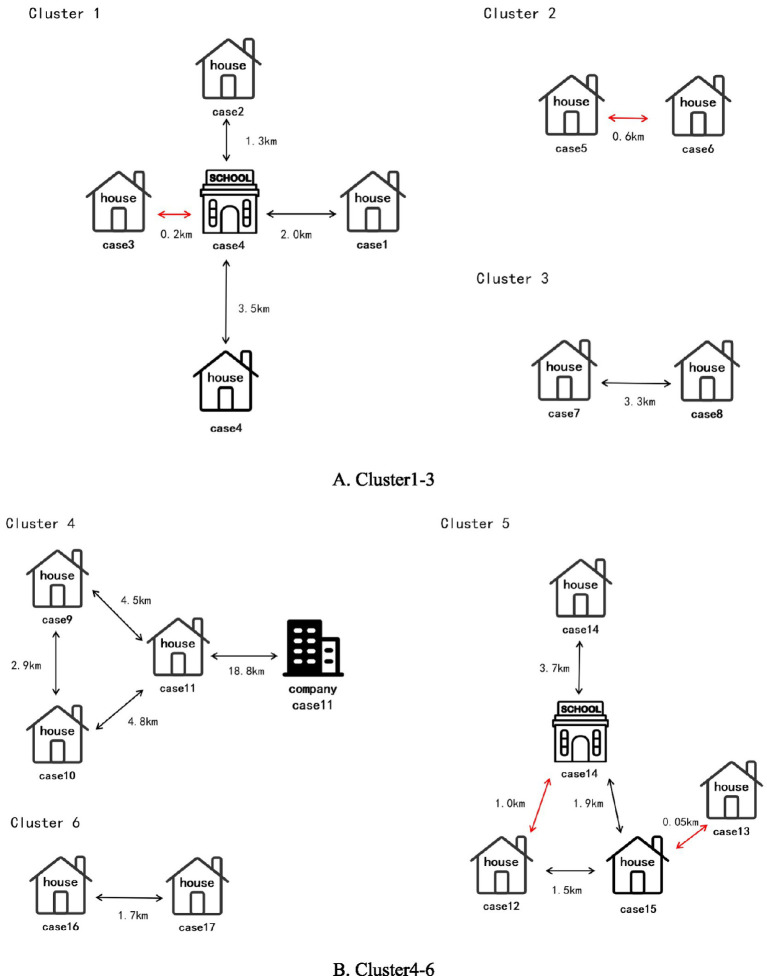
De-identified spatial relationships within genomic clusters (**A**, Clusters 1–3; **B**, Clusters 4–6). House, school, and company icons denote a case’s residence, school, and workplace; the same label appearing more than once is the same patient at different settings, indicating possible exposure outside the home. Values are approximate straight-line distances (km); no real addresses or coordinates are shown. Red arrows indicate case pairs in close proximity (≤1 km).

**Figure 4:**
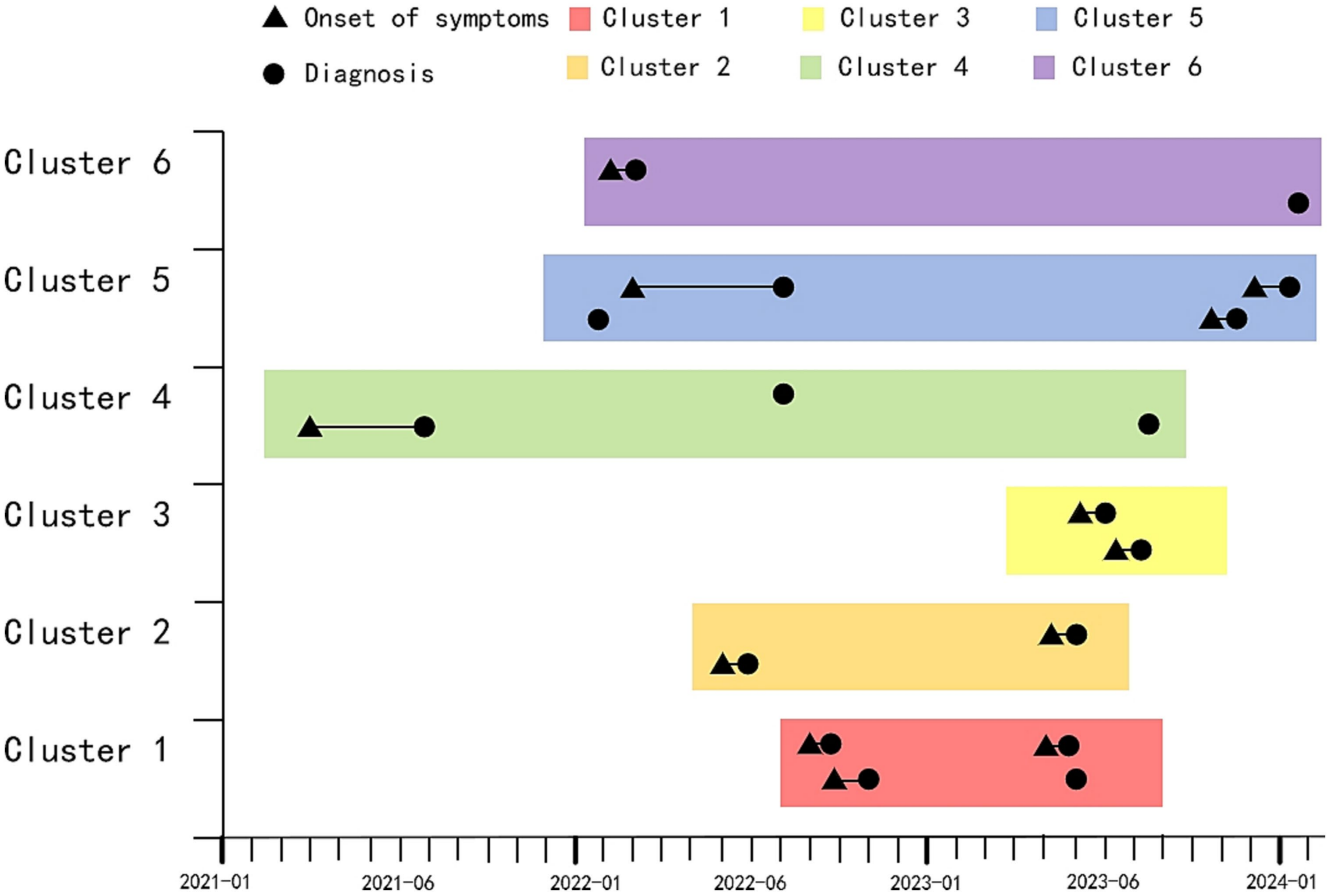
Temporal distribution of clustered cases. Triangles (▲) represent the date of symptom onset, and circles (●) represent the date of diagnosis for each case. Colored horizontal bars indicate the overall time span of each genomic cluster.

To further characterize these relationships, de-identified spatiotemporal analyses were performed (Figures 3A,B; Figure 4). Most clustered cases resided within short distances of one another—several student–non-student pairs within 1 km—with cases in the same cluster developing disease over intervals ranging from months to about 2 years, consistent with the prolonged latency of TB. These patterns indicate marked spatial proximity and temporal aggregation, although they cannot, on their own, establish direct transmission.

**Figure 5:**
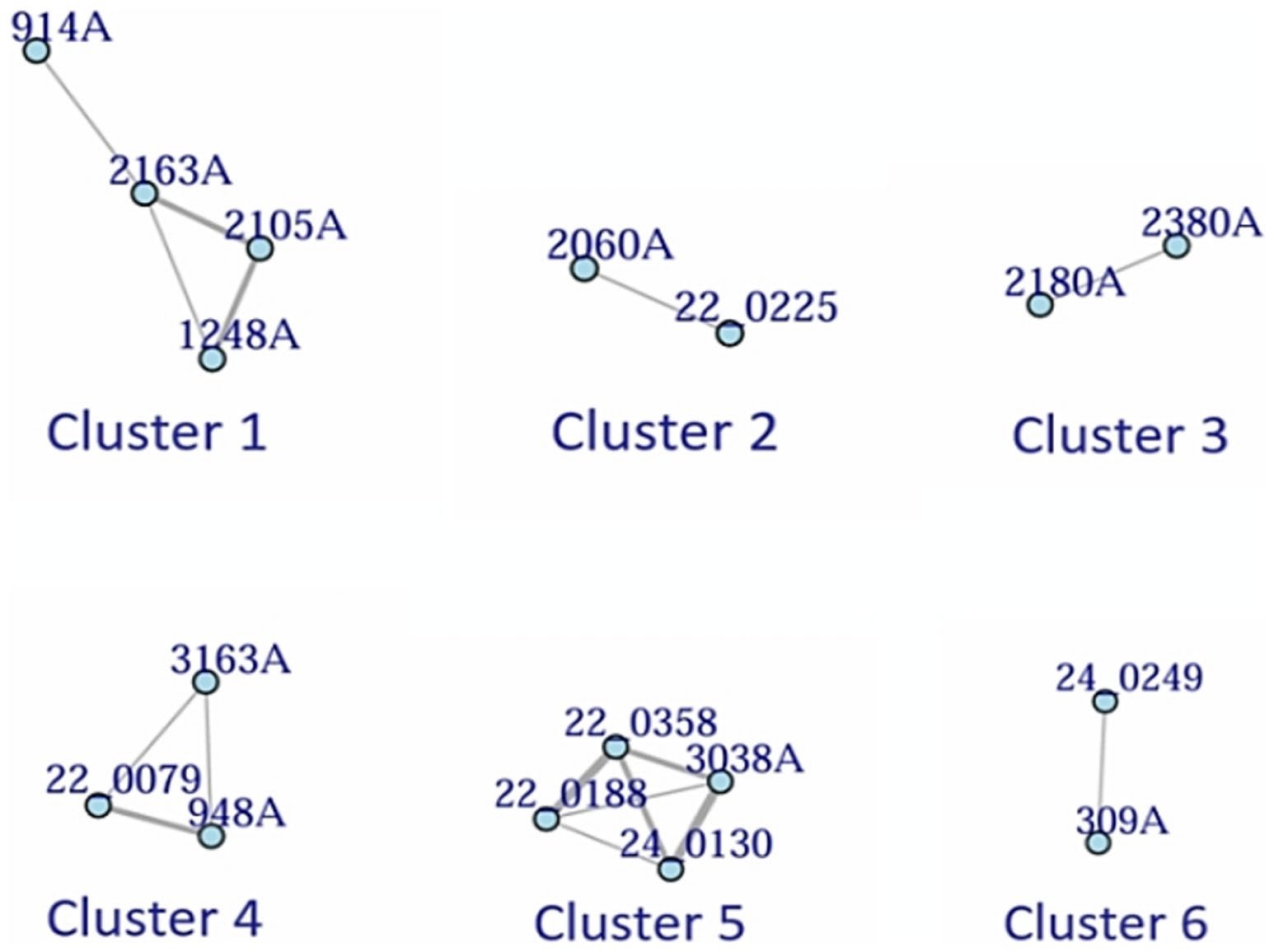
Putative recent transmission network of genetically clustered *Mycobacterium tuberculosis* isolates. The network is based on genomic clustering analysis (≤12 SNP threshold) and epidemiological data. Nodes represent unique patient cases, annotated with their laboratory submission IDs. Edges represent inferred direct transmission events between cases. The width of each edge is proportional to the supporting evidence for transmission, integrating genetic proximity and spatiotemporal overlap.

Based on pairwise genetic distances, we inferred 16 potential recent transmission events (Figure 5). Epidemiological investigation showed no self-reported acquaintance or direct close contact between Case 13 (ID: 220358) and Case 15 (ID: 220188) in Cluster 5. However, they resided in adjacent buildings within the same community, at a straight-line distance of less than 50 meters. Combined with high genomic relatedness, this shared residential environment was consistent with a probable transmission link, with Case 15 representing a potential source case. Of the 16 inferred transmission events, four (25%) involved student cases, all of which represented student-to-non-student connections. Although no direct social contact or common exposure history was identified for these pairs, the possibility of unobserved links cannot be excluded. For the remaining events suggested by genomic proximity, definitive transmission relationships could not be confirmed due to a lack of supporting epidemiological evidence.

### Analysis of risk factors for recent transmission

To identify factors associated with recent TB transmission, we performed univariate and multivariate logistic regression analyses of demographic and clinical characteristics from 170 patients (Table 3). Univariate analysis showed that age was significantly associated with genomic clustering (*p* < 0.05), while occupation demonstrated a borderline non-significant association (*p* = 0.064). In the multivariable Firth penalized logistic regression model, age remained independently associated with genomic clustering. Compared with the reference group (≥45 years), the odds of being in a transmission cluster were significantly higher in the 15–24-year (aOR = 42.75; 95% CI: 5.79–315.50; *p* < 0.001) and 25–44-year (aOR = 18.06; 95% CI: 3.76–86.73; *p* < 0.001) age groups. Sex, student status, household registration status, and treatment history were not independently associated with genomic clustering after adjustment. Consistent with the distribution of clustered cases, the 25–44-year group accounted for the largest number of clustered cases (*n* = 9), followed by the 15–24-year group (*n* = 6).

**Table 3:** Logistic regression analysis of factors associated with clustering among tuberculosis patients with sequencing data in Xuhui District.

Variable	Clustered	Un-clustered	Total	Univariate analysis		Multivariate analysis	
	(*n* = 17)	(*n* = 153)	(*n* = 170)	cOR (95% CI)	*p* value	aOR(95% CI)	*p* value
Sex							
Male	9 (8.2)	101 (91.8)	110	0.58 (0.21, 1.59)	0.289	1.23 (0.40, 3.74)	0.717
Female	8 (13.3)	52 (86.7)	60				
Age in years							
15–24	6 (37.5)	10 (62.5)	16	33.90 (6.03, 190.46)	<0.001	42.75 (5.79, 315.50)	<0.001
25–44	9 (23.1)	30 (76.9)	39	16.95 (3.48, 82.64)	<0.001	18.06 (3.76, 86.73)	<0.001
≥45	2 (1.7)	113 (98.3)	115	Ref		Ref	
Occupation							
Student	3 (30.0)	7 (70.0)	10	–	0.064	0.57 (0.08, 4.05)	0.576
Non-student	14 (8.8)	146 (91.2)	160				
Household registration							
Migrant	8 (13.6)	51 (86.4)	59	1.78(0.65, 4.88)	0.264	0.60 (0.18, 1.99)	0.402
Local resident	9 (8.1)	102 (91.9)	111				
History of tuberculosis							
New case	17 (10.8)	141 (89.2)	158	–	0.240	1.69 (0.08, 35.5)	0.736
Retreatment case	0 (0.0)	12 (100.0)	12				
Strain lineage							
L2	17 (11.1)	136 (88.9)	153	–	0.224		
Non-L2	0 (0.0)	17 (100.0)	17				
Drug resistance							
Yes	3 (6.5)	43 (93.5)	46	0.55 (0.15, 2.01)	0.565		
No	14 (11.3)	110 (88.7)	124				
Chest cavitation							
Yes	5 (9.1)	50 (90.9)	55	0.86 (0.29, 2.57)	0.785		
No	12 (10.4)	103 (89.6)	115				

* Data are *n* (%). For univariate comparisons, Fisher’s exact test was used when expected cell counts were <5 or zero. A dash (“–”) indicates the OR was not estimated (zero-cell counts, or rows analyzed by Fisher’s exact test without a regression estimate). Multivariable analysis used Firth’s penalized logistic regression due to small clustered cases (*n* = 17) and sparse data.

Beyond relative risk, we calculated the population attributable fraction (PAF) to evaluate the impact of different age groups on the overall transmission burden. Individuals aged 15–24 and 25–44 years yielded substantial independent PAFs of 79.71 and 79.65%, respectively. Collectively, the joint PAF for the 15–44 age group reached 88.69%, indicating that these younger cohorts drove the vast majority of genomic clustering in this setting. The age association was robust in a parsimonious sensitivity model (Supplementary Table S2).

## Discussion

Understanding the socio-demographic disparities and health-system gaps that sustain residual TB transmission in low-incidence metropolitan settings is essential for guiding equity-oriented public health interventions. By integrating WGS, demographic data, and field epidemiology, this study provides a detailed characterization of TB transmission patterns in Xuhui District, Shanghai—a densely populated, rapidly urbanizing area with high cross-district mobility. Despite the district’s low overall TB burden, the identification of genomic clusters and analysis of key demographic patterns offers valuable insights into TB control in high-density urban environments.

Notably, while individuals aged 15–24 years showed the highest relative risk, those aged 25–44 years accounted for the largest number of clustered cases. Population attributable fraction analysis suggested that adults aged 15–44 years together contributed nearly 90% of ongoing TB transmission, indicating that young and middle-aged adults jointly drive local transmission rather than only adolescent students. Younger age emerged as the strongest independent predictor of genomic clustering after adjustment for sex, occupation, household registration status, and treatment history, a finding consistent with observations in rapidly urbanizing regions of China (23), Japan (24), and England (25). In Xuhui District, this demographic trend is further shaped by the intersection of age and residency status. While older local residents predominantly represent endogenous reactivation, the younger population—characterized by broader social networks spanning schools, workplaces, and recreational venues—appeared to be the primary driver of recent transmission chains (26). However, beyond these behavioral and social-network factors, the observed age effect may also partially reflect unmeasured socioeconomic and housing-related conditions—such as household crowding and rental instability—which disproportionately affect younger migrants in urban Shanghai.

Importantly, even after adjustment for the measured demographic and clinical confounders (sex, occupation, household registration, and treatment history), this association remained evident. Nevertheless, the modest number of clustered cases identified in this study resulted in relatively wide confidence intervals around the adjusted estimates. Therefore, although younger age remained a robust independent predictor of clustering, the magnitude of the observed associations should be interpreted with caution and confirmed in larger cohorts. In the context of Shanghai’s high-density urban core, younger individuals often serve as “transmission bridges,” linking disparate social circles through intense indoor activities and delayed care-seeking behaviors.

Although no school-wide outbreak was confirmed epidemiologically, genomic and spatial analyses revealed that half of the clusters included at least one student case, and the residential proximity of student–non-student pairs was more consistent with a school–community interface characteristic of dense urban environments (27) than with isolated school-based outbreaks. Consistent with this, clustered student and non-student cases resided in close proximity within shared neighborhoods and developed disease over intervals of months to about 2 years—patterns suggestive of shared environmental exposure or casual community contact, although direct transmission pathways could not be confirmed from the available data. These findings underscore the importance of strengthening linkages between school-based screening and community health services to more effectively control TB transmission in such highly integrated urban landscapes.

The high predominance of lineage 2 (L2) strains (90%) in this study aligns with the long-term endemicity of this lineage in eastern China, which has been associated with enhanced transmissibility (28–30). Despite this biological propensity for transmission, the 10.0% genomic clustering rate observed in Xuhui District indicates modest, localized recent transmission rather than widespread epidemic spread. Taken together, our findings highlight the cryptic, spatially dispersed nature of TB transmission in this low-incidence urban setting, particularly among younger populations with high mobility. The potential school–community interface observed in several genomic clusters highlights the importance of integrated, cross-sectoral interventions that combine enhanced school-based screening, community surveillance, and targeted contact investigations. Such integrated strategies—linking school-based screening, community surveillance, and cross-district data sharing—directly address the socio-demographic disparities and structural gaps that characterize TB control in rapidly urbanizing low-incidence cities.

Regarding drug resistance, fluoroquinolone resistance mutations were identified in 12.4% (21/170) of isolates. This prevalence is moderately higher than the 7.4–8.4% reported in recent population-based genomic surveillance across Shanghai (14, 27). The elevated FQ resistance in our cohort may partly reflect the characteristics of culture-confirmed cases included in genomic surveillance and the local circulation of resistant strains. Furthermore, the 46.2% pre-XDR-TB rate among MDR/RR-TB strains highlights a critical challenge for local TB control. These findings suggest that in high-density urban settings, high FQ resistance might be driven by both prior community-level exposure and localized transmission, necessitating enhanced genomic drug susceptibility testing to prevent the spread of highly resistant clones.

This study has several limitations. First, only culture-positive pulmonary TB cases with viable isolates were analyzed—about one-quarter of all notified cases—because smear-negative, molecularly-confirmed-only, and culture-failed cases cannot be sequenced. Although included and excluded cases were comparable across measured characteristics (Supplementary Table S1), paucibacillary disease—more common in younger people and students—is under-represented, and incomplete district-level sampling may truncate cross-district chains and miss intermediary cases (31); the 10.0% clustering rate should therefore be regarded as conservative.

Second, genomic relatedness indicates recent transmission but cannot establish direct or directional links, and partial discordance between genomic and epidemiological data is common in such studies (32, 33). The proposed school–community interface and the inferred events rest on genomic clustering and spatial–temporal proximity rather than documented contact; confirmed links relied on self-reported acquaintance, and individual activity trajectories and social-contact networks were unavailable, limiting confirmation of transmission pathways.

Third, contact-history investigation was restricted to the 6 months before diagnosis, shorter than the potential multi-year latency of TB; transmission from earlier or intermittent exposures may have been missed, and the window may bias cluster detection toward younger, more socially active cases—relevant when interpreting the observed age distribution.

Finally, several characteristics were measured coarsely: residency was classified by household registration (hukou), an imperfect proxy for mobility and socioeconomic position, and routine surveillance data lacked information on HIV, socioeconomic status, and other risk factors, leaving residual confounding. Given the small number of clustered cases (*n* = 17), the multivariable model is also susceptible to overfitting and high variance; the adjusted odds ratios and PAFs should therefore be regarded as exploratory, and although a parsimonious sensitivity analysis yielded a consistent age effect (Supplementary Table S2), these estimates require validation in larger cohorts.

## Conclusion

This study demonstrates that younger and middle-aged adults are the primary drivers of recent TB transmission in low-incidence metropolitan settings. While adolescents and young adults face the most acute individual risk, the broader working-age population contributes more substantially to the population-level burden. Our findings suggest that precision TB control in high-density urban areas may benefit from a combined approach: maintaining robust school-based screening while expanding surveillance to integrate social and occupational networks of the active workforce. The potential school–community interface observed in several genomic clusters highlights the importance of integrated, cross-sectoral interventions linking school health programs and community-based TB control. Institutionalizing genomic epidemiology alongside traditional surveillance may facilitate identification of otherwise unrecognized transmission chains and support TB elimination targets in urban China.

## Data Availability

The data analyzed in this study are subject to the following licenses/restrictions. The datasets are subject to two main restrictions: patient privacy protection: the raw epidemiological and clinical data contain de-identified but potentially identifiable information of tuberculosis patients. Therefore, they cannot be publicly shared to protect personal privacy in accordance with China’s Personal Information Protection Law. Institutional data governance: the data belong to the routine public health surveillance records of the Shanghai Xuhui District Center for Disease Control and Prevention. Access is governed by the center’s institutional data management policies. Data access requires formal review and approval by the Center and can be granted only for legitimate research purposes upon reasonable request to the corresponding author. The *Mycobacterium tuberculosis* whole-genome sequencing data reported in this paper have been deposited in the Genome Sequence Archive (GSA) at the National Genomics Data Center, Chinese Academy of Sciences, under accession number CRA037559. These data are publicly accessible at https://ngdc.cncb.ac.cn/gsa and are available without restrictions. Requests to access these datasets should be directed to MY (ymeixia@hotmail.com).

## Footnotes

1
https://itol.embl.de/
