# Supplementary material for: Transmission dynamics and attributable burden of tuberculosis among young and middle-aged adults in urban Shanghai: a genomic epidemiology study
Source: Front Public Health. 2026 Jul 8;14:1868508. doi: 10.3389/fpubh.2026.1868508 (PMC13388792; doi:10.3389/fpubh.2026.1868508)
Supplement: Supplementary file 1 [file Table_1.DOCX]

**Supplementary Table S1.** Baseline characteristics of included (n=170) vs. excluded (n=471) TB patients in Xuhui District

|  | **Included（n=170）** | **Excluded（n=471）** | ***χ*^2^** | ***p* value** |
| --- | --- | --- | --- | --- |
| **Sex** |  |  |  |  |
| Male | 110（64.71） | 292（62.00） | 0.392 | 0.531 |
| Female | 60（35.29） | 179（38.00） |  |  |
| **Age in years** |  |  |  |  |
| 15-24 | 16（9.41） | 43（9.13） | 1.071 | 0.784 |
| 25-44 | 39（22.94） | 126（26.75） |  |  |
| 45-64 | 44（25.88） | 121（25.69） |  |  |
| ≥65 | 71（41.76） | 181（38.43） |  |  |
| **Treatment history** |  |  |  |  |
| New case | 158（92.94） | 415（88.11） | 3.074 | 0.080 |
| Retreatment case | 12（7.06） | 56（11.89） |  |  |
| **Household registration** |  |  |  |  |
| Migrant patients | 59（34.71） | 155（32.91） | 0.181 | 0.671 |
| Resident patients | 111（65.29） | 316（67.09） |  |  |
| **Occupation** |  |  |  |  |
| Students | 10（5.88） | 30（6.37） | 0.051 | 0.822 |
| Non-students | 160（94.12） | 441（93.63） |  |  |
