# Supplementary material for: Transmission dynamics and attributable burden of tuberculosis among young and middle-aged adults in urban Shanghai: a genomic epidemiology study
Source: Front Public Health. 2026 Jul 8;14:1868508. doi: 10.3389/fpubh.2026.1868508 (PMC13388792; doi:10.3389/fpubh.2026.1868508)
Supplement: Supplementary file 2 [file Table_2.DOCX]

***Supplementary Table S2.*** *Sensitivity analysis of the association between age and genomic clustering using a parsimonious Firth penalized logistic regression model
(Adjusted for sex and student status only)*

| Variable | aOR (95% CI) | *P* value |
| --- | --- | --- |
| **Sex** |  |  |
| Male | 1.34 (0.45, 4.01) | 0.598 |
| Female | Ref | Ref |
| **Age in years** |  |  |
| 15–24 | 38.04 (5.64, 256.58) | <0.001 |
| 25–44 | 15.35 (3.45, 68.29) | <0.001 |
| ≥45 | Ref | Ref |
| **Occupation** |  |  |
| Student | 0.69 (0.10, 4.58) | 0.699 |
| Non-student | Ref | Ref |

*Note: The parsimonious model included only age group, sex, and occupation to assess the robustness of the age effect against model overfitting. The results were consistent with the main multivariable model, supporting the primary finding that younger age is associated with genomic clustering even under reduced adjustment.*
